# Novel Molecular Therapeutics Targeting Signaling Pathway to Control Hepatitis B Viral Infection

**DOI:** 10.3389/fcimb.2022.847539

**Published:** 2022-02-18

**Authors:** Yan Yan, Yuanwang Qiu, Chantsalmaa Davgadorj, Chunfu Zheng

**Affiliations:** ^1^ Laboratory for Infection and Immunity, Hepatology Institute of Wuxi, The Fifth People’s Hospital of Wuxi, Affiliated Hospital of Jiangnan University, Wuxi, China; ^2^ Department of Microbiology, Immunology and Infectious Diseases, University of Calgary, Calgary, AB, Canada

**Keywords:** HBV, infection, TLR, signaling pathway, carcinogenesis, therapeutics

## Abstract

Numerous canonical cellular signaling pathways modulate hepatitis B virus (HBV) replication. HBV genome products are known to play a significant role in regulating these cellular pathways for the liver’s viral-related pathology and physiology and have been identified as the main factor in hepatocarcinogenesis. Signaling changes during viral replication ultimately affect cellular persistence, multiplication, migration, genome instability, and genome damage, leading to proliferation, evasion of apoptosis, block of differentiation, and immortality. Recent studies have documented that numerous signaling pathway agonists or inhibitors play an important role in reducing HBV replication *in vitro* and *in vivo*, and some have been used in phase I or phase II clinical trials. These optional agents as molecular therapeutics target cellular pathways that could limit the replication and transcription of HBV or inhibit the secretion of the small surface antigen of HBV in a signaling-independent manner. As principle-based available information, a combined strategy including antiviral therapy and immunomodulation will be needed to control HBV infection effectively. In this review, we summarize recent findings on interventions of molecular regulators in viral replication and the interactions of HBV proteins with the components of the various targeting cellular pathways, which may assist in designing novel agents to modulate signaling pathways to prevent HBV replication or carcinogenesis.

## Introduction

Hepatitis B virus (HBV) is a DNA virus with a partially double-stranded DNA genome that is circular and contains ~3.2 kb genome in length, encoding seven different proteins, such as HBV surface antigen (HBsAg), HBV core antigen (HBcAg), HBV e antigen (HBeAg), and the transcriptional transactivator HBV X protein (HBx), which controls HBV transcription from covalently closed circular DNA (cccDNA) (Tu et al., 2017). As many as 887,000 deaths are caused by HBV infection worldwide each year (Revill et al., 2019). HBV infection is very different from other viral infections. HBV is a “stealth virus” that induces negligible immune responses in the initial stages of infection, involving type I interferons (IFN-I) (Wieland et al., 2004). Patients with chronic hepatitis B (CHB) infection generally begins with an asymptomatic non-inflammatory (or immune tolerant) phase and poorly-activated HBV-specific CD8^+^ T cells (Tu et al., 2017; Yan et al., 2021), while the hepatitis C virus (HCV) can stimulate IFNs early in infection (Su et al., 2002). Chronic HBV infection is a major global public health threat, with more than 250 million people worldwide, causing almost 40% of liver cancer (hepatocellular carcinoma, HCC) (Meng et al., 2019; Revill et al., 2019; Lee et al., 2021). The incidence of liver cancer in patients with chronic HBV infection is usually 10-25 times that in non-infected patients (Tang et al., 2018). The recent “Global Burden of Disease” study highlights that the total number of viral hepatitis deaths, including HCC, now exceeds the number of deaths from tuberculosis, HIV/AIDS, and malaria (Collaborators, 2017).

Currently recommended therapeutic agents for the treatment of chronic HBV infection, including current agents, nucleos(t)ide analogs (NAs), and pegylated-interferon alfa (peg-IFN-α), have been approved to inhibit HBV DNA replication, which can interrupt or prevent the risk of developing cirrhosis and hepatocellular carcinoma (European Association for the Study of the Liver, 2017). However, both of them have limitations. The NAs have little effect on the cccDNA pool, and the loss of HBsAg (functional cure) is rare. For the peg-IFN-α, the processing time is limited and acts at different stages of the HBV life cycle. However, only people with certain conditions have a good response rate in HBsAg loss (Brouwer et al., 2019). Because cccDNA is difficult to clear and may cause recurrence, experts recommend that monitoring disease progression and HCC risk in all patients is still necessary (European Association for the Study of the Liver, 2017). As immunomodulatory drugs, including compounds that block HBV from entering hepatocytes, prevent cccDNA amplification and viral spread; Other compounds that affect core assembly, drugs that target HBV RNase-H, interfere with RNA molecules and nucleic acid polymers may be interventions in the viral life cycle (Alexopoulou et al., 2020). Traditional therapeutic vaccines trials have not shown ideal effects in HBV chronically infected individuals (Michel and Tiollais, 2010), so a “functional cure” is currently an ideal state for treatment with peg-IFN-α, characterized by the complete loss of HBsAg regardless of the presence or absence of anti-HBs antibodies. Nevertheless, recent studies have shown that less than 10% of people achieve the desired clinical HBV cure (Meng et al., 2019). Thus now, we also need to find more effective therapeutics.

Future therapeutic strategies are being studied to achieve a “cure” for the diseases, such as combining drugs that target the viral life cycle. The underlying mechanism of HBV replication is multifactorial. Research and practice have demonstrated that HBV targets innate immune signaling pathways to evade or inhibit the host’s antiviral responses (Luangsay et al., 2015; Cheng et al., 2017; Tu et al., 2017; Meng et al., 2019). In addition, viral gene expression inhibits signaling pathways during HBV replication, such as Toll-like receptor (TLR)-signaling cascade (Vincent et al., 2011; Luangsay et al., 2015). For example, HBsAg, HBeAg, and HBV virions were found to contribute to reducing the expression of TLR-induced antiviral activity and impairing the production of tumor necrosis factor-a (TNF-α), interferons-α (IFN-α), and pro-inflammatory cytokines in hepatocyte lines, associated with the activation of interferon-regulated transcription factor 3 (IRF3) in hepatic non-parenchymal cells (NPCs), nuclear factor kappa B (NF-κB), and extracellular signal-modulated kinase (ERK)1/2 of transcription factor reduction. (Vincent et al., 2011; Huang et al., 2015; Cheng et al., 2017). TNF-α and IFN-I play an essential role in killing virus-infected hepatocytes in the process of eradicating HBV (Lu et al., 2021). The innate immune system recognizes pathogens *via* pattern recognition receptors (PRR), including TLRs and retinoic acid-inducible gene I (RIG-I)-like receptors (RLRs) or checkpoint inhibitor stimuli. Studies have shown that it is necessary to rely on cytokines and chemokines in tissue cells to recruit active immune cells (CD8^+^ T cells) into the appropriate immune microenvironment to regulate Ag-specific apoptosis and virus antigen degradation, which helps to eliminate HBV-infected and damaged hepatocytes, like IFN-α (Li et al., 2016; Zhang et al., 2021). When Ag-specific apoptosis and viral antigen degradation occur, it will drive Ag-presenting cells to induce inflammation and cytokines by modulating cell signaling pathways and cell cycles (Cheng et al., 2017).

Therefore, to study the roles of cellular signaling pathways and their associated intervention agents in modulating HBV replication, like the TLR signaling pathways, chemokine receptor (CKR) signaling pathways, and the phosphatidylinositol 3-kinases/protein-serine-threonine kinase (PI3K/AKT), RAS/MEK/ERK, Janus family tyrosine kinases/signal transducer and activators of transcription (JAK/STAT) signaling pathways, will help provide valuable information on the effectiveness of individualized therapeutic approaches by inhibiting or restoring signaling functions.

## TLR Agonist-Mediated Therapeutics for Controlling HBV Replication

The outcome of HBV infection is influenced by the early viral interactions with hepatocyte innate immune responses, but the study on early interactions of viral infections remains very limited. On the other hand, few data are obtained from acute infection models that indicate that activation of innate reactions during natural HBV infection is predominantly weak or absent (Luangsay et al., 2015). Previous studies have demonstrated that TLRs play an important role in viral recognition and inducing appropriate immune responses. Therefore, TLRs can be considered key sensors for inducing immune responses against HBV (Bagheri et al., 2014). The TLR subgroups of TLR3, TLR7, TLR8, and TLR9 recognize nucleic acids, particularly viral DNA/RNA, while the other subgroups detect bacterial, fungal cell wall components, and some viral proteins (Xiaoyong et al., 2012). Thus, small synthetic molecules can mimic TLR ligands, activate TLR downstream signal pathways and inhibit HBV replication.

**Figure 1:**
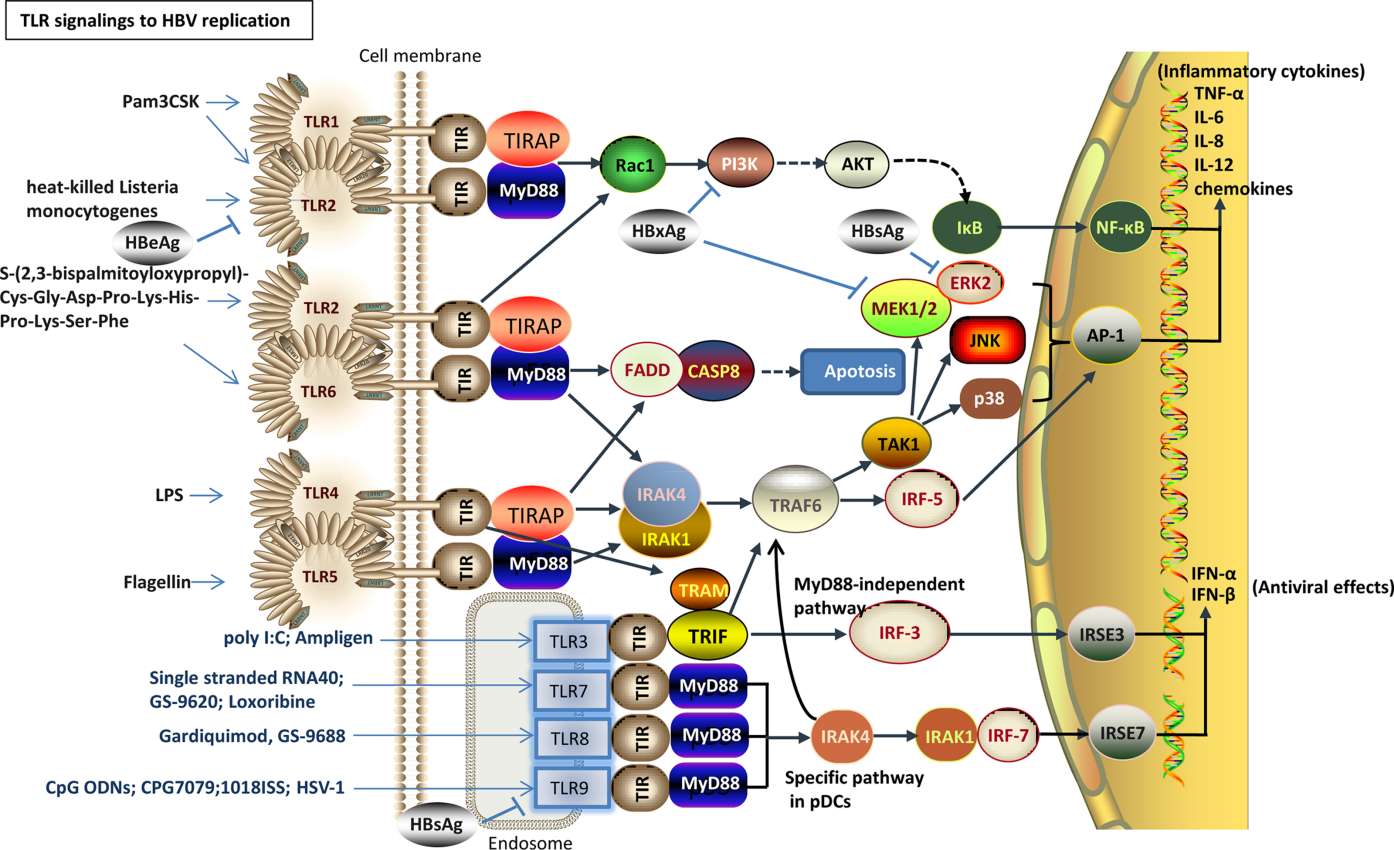
TLR agonists as promising agents against HBV infection. After the agonist activates the corresponding TLR signaling pathway, downstream signaling molecules are activated, such as PI3K/AKT, MEK1/2, ERK2, JNK, NF-κB or IRF3, promote the antiviral cytokine IFN-α/β and the secretion of pro-inflammatory or inflammatory cytokines IL-6, IL-8, IL-12, and chemokines, and promote anti-HBV replication. HBsAg and HBxAg inhibit the activity of the TLR downstream signaling molecules.

TLR7, TLR8, and TLR9 agonists have been used in phase I or phase II clinical trials to treat chronic HBV infection (Xiaoyong et al., 2012; Ma et al., 2015). In the preclinical study, TLR7 agonists were found to induce sustained anti-HBV activity in AAV/HBV mice *via* non-cytolytic mechanisms (Herschke et al., 2021). The stimulation of TLR7 agonist has a similar effect on serum viral DNA and Ags clearance in the chimpanzee and woodchuck models of CHB (Menne et al., 2015; Li et al., 2018). In a clinical study, TLR7 was an activator of innate and adaptive immune responses, Vesatolimod (GS-9620) acted as an oral agonist for TLR7, and an oral antiviral regimen similar to TLR8 study lasted up to 12 weeks. However, serum IFN-a was not significantly expressed. The results showed that HBsAg was not significantly reduced despite patients exhibiting targeted biomarker responses and viral suppression (Janssen et al., 2018). At the same time, TLR8 is an endosomal innate immune receptor that can be used as a target for the treatment of viral infections. *In vivo* studies have shown that Selgantolimod is a novel TLR8 agonist that plays a more important role in inducing the antiviral response for the treatment of CHB, inducing 40% curative treatment in a woodchuck CHB model (Daffis et al., 2017). Furthermore, Selgantolimod-induced human PBMC cytokines help reduce viral parameters in HBV-infected human hepatocytes (Mackman et al., 2020). The clinical study investigated the safety, tolerability, and pharmacokinetics of selgantolimod in healthy volunteers. Selgantolimod induces an immediate dose-dependent increase in serum antiviral cytokines, chemokines, and acute-phase proteins, which is important for the expansion and activity of T-cell subsets and innate immunity (Reyes et al., 2020). *In vivo*, a weekly dose of oral selgantolimod induced a dose-dependent increase in serum IL-12p40 and IL-1 receptor antagonists in cynomolgus monkeys, which responded similarly in healthy volunteers (Reyes et al., 2020). TLR9 recognizes DNA sequences from bacteria or viruses in the form of unmethylated cytidine phosphate guanosine (CpG) motifs (Ruslan and Charles, 2000). In addition, studies have shown that specific antiviral treatment against HBV can help restore the function of TLRs in chronic HBV infection (Zhang and Lu, 2015), except for lamivudine (Chin et al., 2008). Combination therapy, including the TLR9 agonist [CpG oligodeoxynucleotides (ODNs)] and entecavir, induce early antiviral responses and enhance inhibition of viral replication in a woodchuck model of chronic hepadnaviral infection, but neither of these agents can be used alone, suggesting that they are synergistic (Meng et al., 2016). The other two TLR9 agonists of class B CpG ODNs are called CPG 7909, and 1018 ISS combined with HBsAg to induce high titers of HBsAb with higher affinity effective in immune-compromised individuals (Cooper et al., 2004; Curtis and David, 2011). The effects of the TLR9 agonists CPG-ODNs are restricted to plasmacytoid dendritic cells (pDCs) and B lymphocytes (Vincent et al., 2011). In CHB patients, TLR7/9 agonists help promote the production of IFN-α from patients’ pDCs (Vincent et al., 2011; Xu et al., 2012) (**Table 1** and **Figure 1**).

**Table 1:** TLR agonists as clinical molecular therapeutics for controlling HBV replication.

TLR	Agonist	Immune response type	Adaptor	Responsive cytokine and cell	Model	Clinical phase	Reference
**TLR1**	palmitoyl-3-cysteine-serine-lysine-4 (Pam3CSK)	innate	MyD88	TNF-α, IL10, and IL-6↑	C57BL/6 and MyD88 mice	preclinical	(Wu et al., 2007; Xiaoyong et al., 2012; Jiang et al., 2014)
**TLR2**	Pam3CSKL; S-(2,3-bispalmitoyloxypropyl)- Cys-Gly-Asp-Pro-Lys-His-Pro-Lys-Ser-Phe	innate and adaptive	MAPK, PI3K/AKT, IRF3, MyD88, NF-κB and ERK1/2	IL-6, TNF-α, IFNs, IL-1β↑; CD4^+^ and CD8^+^ T cells↑	Chronic and acute HBV infected mouse; C57BL/6 and MyD88 mice	preclinical	(Wu et al., 2007; Wu et al., 2009; Huang et al., 2015; Xu et al., 2017; Lin et al., 2018)
**TLR3**	polyinosine-polycytidylic acid poly(I:C); Ampligen	innate	TRIF	TNF-α, IL-6, IL10 and IFN-β↑	KCs, LSECs of mice and HBV-Met cells; C57BL/6 and MyD88 mice	preclinical	(Niu et al., 1993; Wu et al., 2007; Xiaoyong et al., 2012; Jiang et al., 2014)
**TLR4**	Lipopolysaccharide (LPS)	innate	MAPK and PI3K/AKT	TNF-α, IL-6, IL10, and IFN↑	WHV; C57BL/6, and MyD88 mice	preclinical	(Wu et al., 2007; Wu et al., 2009; Jiang et al., 2014)
**TLR5**	*Salmonella typhimurium* flagellin	innate	MyD88	TNF-α, IL-6, and IL-10↑;	C57BL/6 and MyD88 mice	preclinical	(Wu et al., 2007; Xiaoyong et al., 2012; Jiang et al., 2014; Yan et al., 2020)
**TLR6/2**	S-(2,3-bispalmitoyloxypropyl)- Cys-Gly-Asp-Pro-Lys-His-Pro-Lys-Ser-Phe	innate	MyD88	TNF-α and IL-6↑	C57BL/6 and MyD88 mice wild-type and MyD88 mice	preclinical	(Wu et al., 2007)
**TLR7**	Single-stranded RNA40; GS-9620 (Vesatolimod); loxoribine	adaptive	–	type I/II IFN↑, IL-6↑; CD8^+^ T cells and B cells↑	Cellular Glycolysis; chimpanzee, WHV; CHB patients’ specimens	clinical phase II	(Jiang et al., 2014; Menne et al., 2015; Janssen et al., 2018; Li et al., 2018; Li et al., 2019b)
**TLR8**	Gardiquimod, GS-9688 (Selgantolimod); ssRNA40	Innate and adaptive	–	IFN↑, CD40↑, CD80↑, CD86↑; IL-21↑; IL-12p40↑; IL-6↑; CD8+ T cells↑	WHV	clinical phase Ia	(Jiang et al., 2014; Reyes et al., 2020; Daffis et al., 2021)
**TLR9**	cytidine phosphate guanosine (CpG) oligodeoxynucleotides (ODNs); HSV-1; CPG7079; 1018ISS	Innate	IRF7	IFN↑, ISGs↑; CD40↑, CD80↑, CD86↑; pro-inflammatory cytokines↑	WHV	clinical phase Ia	(Cooper et al., 2004; Curtis and David, 2011; Vincent et al., 2011; Xu et al., 2012; Meng et al., 2016)

KC, Kupffer cell; LSEC, liver sinusoidal endothelial cell.

In addition to some TLRs that have been found to play an important role in inhibiting HBV replication *in vivo* and are undergoing clinical trials, activation of the TLR3 pathway follows in the production of IFN-β, which also inhibits HBV replication in mouse livers (Honda and Taniguchi, 2006; Broering et al., 2011). TLR2 or TLR4 signaling activates intracellular pathways in hepatocytes and peripheral CD4^+^ T cells, including mitogen-activated protein kinase (MAPK) and PI3K/AKT signaling molecules, and reduces HBV replication in an IFN-independent manner (Wu et al., 2009; Zhang et al., 2009; Zhang and Lu, 2015). For example, the pre-activation of a TLR2 agonist (lipopolysaccharide, LPS) helps enhance the adaptive immune response of CD8^+^ T cells and accelerates HBV clearance in a mouse model (Lin et al., 2018) and woodchuck hepatitis virus (WHV) model (Zhang et al., 2009). In an acute HBV replication mouse model, flagellin protein as a bacterial PAMP plays a pivotal role in regulating intrahepatic CD8^+^ T-cell responses by activating the TLR5 pathway (Yan et al., 2020). TLR6 overcome HBV tolerance in the CHB mouse models by enhancing HBV-specific antibody levels and follicular-assisted T-cell responses (He et al., 2017). This study proposes a novel mechanism by which TLR6 acts on the immune response and a therapeutic approach that breaks down the tolerance of HBV. Therefore, in addition to the agonists that have entered clinical trials, these TLR agonists are promising candidates as immunomodulators or combination therapies for inducing anti-HBV activity in chronic HBV animal models (**Table 1** and **Figure 1**).

## Therapeutics That Modulate RIG-I-Like Signaling Pathways to Control HBV Replication

Acute viral infections usually trigger intracellular innate immune activation, leading to intracellular antiviral defenses. In addition to TLRs, RLRs (RIG-I-like receptors), NOD-like receptors (NDRs), and intracellular DNA sensors cGAS-STING are involved in sensing viral infections (Lee et al., 2021). A study showed that strategies to activate RIG-I signaling helped enhance antiviral defense against HBV, which also meant that RIG-I signal activation could mediate the innate immune system to control HBV replication (Lee et al., 2021). A small-molecule compound, F7, and 5′-triphosphate-poly-U/UC PAMP RNA agonists of RIG-I can suppress the formation of HBV cccDNA and accelerated decay of established cccDNA and are additives to the actions of entecavir. It has been highlighted that activating the RIG-I pathway and IRF3 to induce innate immune action provides therapeutic benefits toward eliminating cccDNA in hepatocytes (Lee et al., 2021). It has been shown that the nuclear factors of activated T cells 3 (NFATc3) inhibit hepatocarcinogenesis and HBV replication by positively regulating RIG-I-mediated IFN transcription (Zao et al., 2021). Together, these findings collectively reveal a novel regulatory signaling cascade, the RIG-I/NFATc3/IFNs axis, which inhibits HBV replication and hepatocarcinogenesis by enhancing the hepatocytes functional axis’s immune responses, which might potentially be exploited for therapeutic benefits in the clinical treatment of HBV-related HCC.

**Figure 2:**
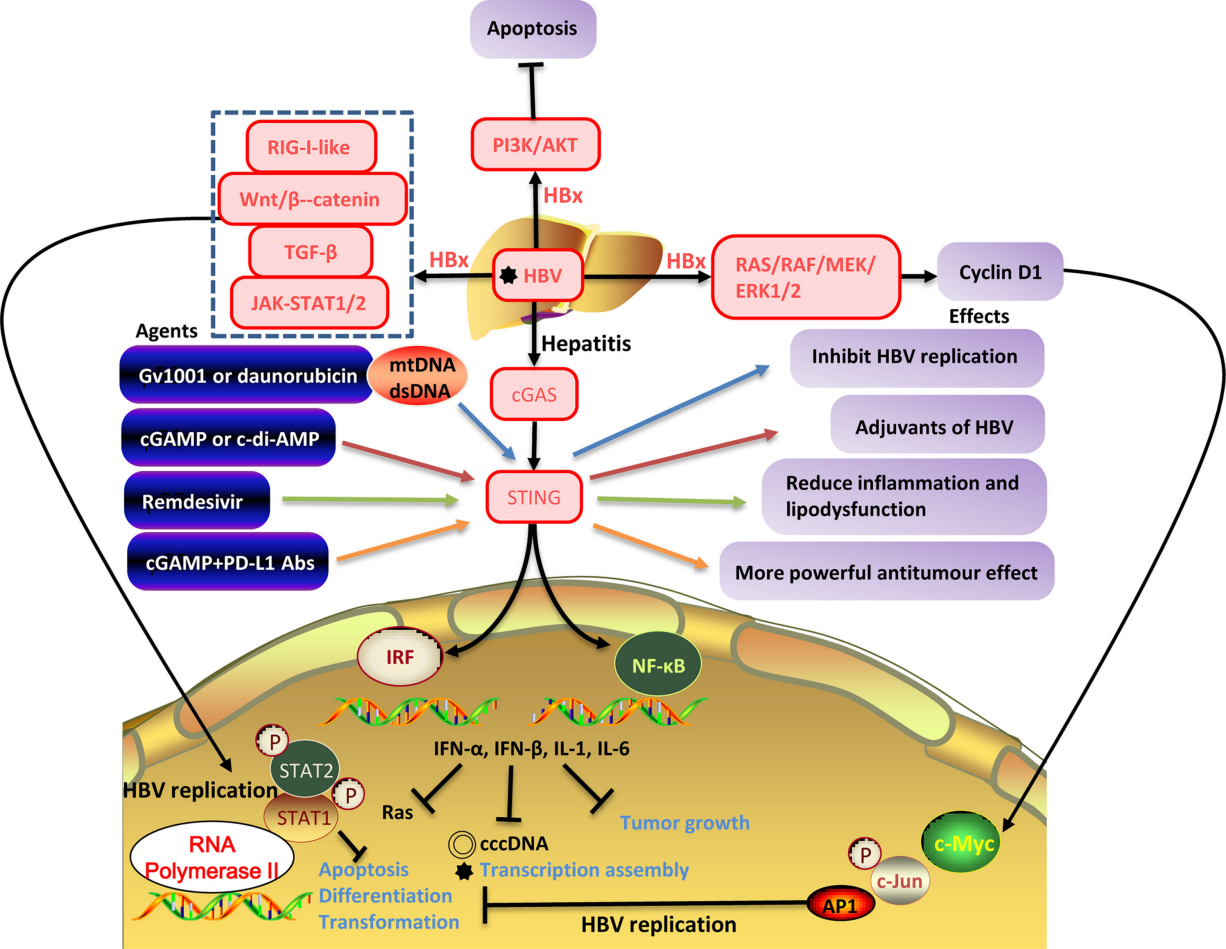
The interaction between HBV replication and the cellular signaling pathways. HBV infection activates downstream RIG-I-like, Wnt/β-catenin, TGF-β, GAS-STING signaling pathways, PI3K/AKT, JAK/STAT, RAS/MEK/ERK, promotes the secretion of antiviral cytokines (IFN-α, IFN-β) and inflammatory cytokines (IL-1, IL-6) inhibits the production of HBV cccDNA, and transcription assembly of viruses inhibits tumor growth. However, HBxAg can activate certain cellular signaling pathways and promote HBV replication. HBV replication inhibits apoptosis, differentiation, and transformation and is closely related to tumorigenesis.

In a clinical trial, Inarigivir (SB 9200) is a RIG-I or RLR agonist that can reduce HBV DNA/RNA, which is more potent than NAs and induces 26% of patients’ HBsAg loss (Smolders et al., 2020). A recent study showed that activated RIG-I-induced transcription factors lead to IFN-I and pro-inflammatory cytokines production. The secreted IFN-I binds to the cognate IFN-α/β receptor (IFNAR) on the surface of most nucleated cells, then initiates JAK/STAT signaling and induces hundreds of ISGs to exert a direct antiviral effect (Zhou et al., 2021). Another *in vivo* study showed that low-dose oral Inarigivir was well tolerated in HBV infected patients and was associated with a decrease in HBV DNA, RNA, and HBsAg (Smolders et al., 2020). In the absence of a fully activated immune response, this effect is visible and consistent with direct antiviral effects that may reflect targeted HBV RNA encapsulation (Yuen et al., 2018) (**Table 2** and **Figure 2**).

**Table 2:** The replication of HBV modulated by cellular signaling pathways.

Signaling pathway	Agent	Target	Model	Clinical phase	Reference
**RIG-I**	MDA5, F7, poly-U/UC PAMP, SB 9200 (Inarigivir)	IRF3	Huh7 cells, HepG2-hNTCP cells	clinical phase II	(Smolders et al., 2020; Lee et al., 2021)
**Wnt/β−catenin**	miR17~92, miR106b~25, Curcumin	DDX5	HBV patients	clinical phase II	(Hesari et al., 2018; Mani et al., 2020)
**TGF-β**	Iron, SMAD7	miR-125a-5p, miR-151-5p	CHB patients	clinical phase I	(Park et al., 2012; Argentou et al., 2016)
**cGAS-STING**	Gv1001, daunorubicin	mitochondrial stress and hepatocyte DNA damage	HepG2 cells, HepG2-2.15 cells, human liver chimeric mice	preclinical	(Imai et al., 2018; Verrier et al., 2018; Choi et al., 2020; Chen et al., 2021a; Chen et al., 2021b)
**PI3K/AKT**	Ly294002; AKTi, Rapamycin, tripeptidyl peptidase II, nicotine	PI3K, murine immature dendritic cells	HepG2 cells, Huh7 cells, HepG2.2.15 cells, HepAD38 cells or HK-2 cells, HBV transgenic mice, and ATG5 knockout HBV transgenic mice	preclinical	(Xiang and Wang, 2018; He et al., 2020) (Tan et al., 2021)
**JAK/STAT**	HBV-miR-3; IFN-CSP; CDM-3008; IFN-λ; betaine, Tapsin	SOCS5; IFNAR2	HepG2-NTCP cells, HepG2.2.15 cells, HLA-A2 transgenic mice. C57BL/6-HBV transgenic mice	preclinical	(Lu et al., 2015; Zhang et al., 2016; Makjaroen et al., 2018; Wu et al., 2018; Furutani et al., 2019; Zhao et al., 2020)
**RAS/MEK/ERK**	Polyguluronate sulfate (PGS), MLN4924, FoxO4	NF-κB and RAF/MEK/ERK, ERK-HNF1α-C/EBPα-HNF4α axis	HepG2.2.15 cells, C57BL/6 mice	preclinical	(Wu et al., 2016; Li et al., 2019a; Xie et al., 2021)
**Chemokine**	CCL19, CXCL13	Innate and adaptive	C57BL/6 mice	preclinical	(Liu et al., 2017; Yan et al., 2021)

## Therapeutics That Modulate Wnt/β-Catenin Signaling Pathways to Control HBV Replication

It is known that viral-associated proteins in the course of viral infection are involved in multiple cellular pathways, including Wnt/β-catenin, transforming growth factor (TGF)-β, and RAF/MAPK, which also regulate replication of the virus itself. This activation process affects cell persistence, reproduction, migration, alteration, and genomic instability (Daud et al., 2017). The previous study has shown that HBV genomic products are involved in the induction of the Wnt/β-catenin signaling pathway, enhancing this pathway and leading to the development of liver cancer (Daud et al., 2017). HBx has been demonstrated to be involved in the initiation and progression of HCC *via* the COX−2/Wnt/β−catenin pathway (Zheng et al., 2018). The interplay of the Wnt/β-catenin pathway and miRNAs (such as miR-26a, miR-15a, miR-16-1, miR-148a, miR-132, miR-122, miR-34a, miR-21, miR-29a, miR-222, and miR-199a/b-3p) in HBV pathogenesis results in HCC (Rana et al., 2019). From an evolutionary perspective, these non-coding RNAs are highly conserved, and the regulation of different genes mediated by miRNAs involves complementary interactions with their mRNAs (Liu et al., 2011). They play a crucial role in basic cellular life processes, from cell proliferation to apoptosis. These HBV-mediated miRNAs may demonstrate future treatment options for HBV-Wnt/β-catenin-associated HCC (Rana et al., 2019). Furthermore, inhibitors of miR17-92 and miR106b-25 (anti-avian) have been shown to restore DDX5 levels, reduce DVL1 expression, and suppress HBV replication and Wnt signaling in HBV-related hepatocellular carcinoma (Mani et al., 2020). Therefore, the Wnt signaling is inextricably linked to HBV replication

Curcumin is a yellow-orange powder derived from the Curcuma longa plant and grown in India, Southeast Asia, and other tropical regions (Tutanov et al., 2016). Curcumin has been extensively used in traditional medicine for centuries. This component is non-toxic and has different therapeutic properties such as anti-inflammatory, anti-cancer, antiviral, antibacterial, anti-fungal, anti-parasites, and anti-oxidants (Hesari et al., 2018). It has been reported to inhibit tumor growth and apoptosis of gastric carcinoma *in vitro* and *in vivo* through inhibition of Wnt/β-catenin signaling and reduced expression of Wnt target genes (Zheng et al., 2017). In a clinical trial, curcumin also showed its therapeutic effect on HBV patients by targeting various cellular and molecular pathways such as Wnt/β-catenin, Ap1, STAT3, MAPK, and NF-κB signaling (Hesari et al., 2018) (**Table 2** and **Figure 2**).

## Therapeutics That Modulate TGF-β Signaling Pathways to Control HBV Replication

It has been defined that the effects of iron or TGF-β-induced TGF-β/BMP signaling in the HepG2 2.2.15 cell model of hepatitis B virus replication. The results showed that iron increased, but TGF-β decreased HBV mRNA expression. Iron or TGF-β alter microRNA expression in the opposite direction, but iron can significantly induce a decrease in HBV replication (Park et al., 2012). On the other hand, chronic liver injury induced by HBV infection includes the development of liver fibrosis, cirrhosis, and liver cancer, and cytokines that regulate the inflammatory response are involved in the induction of such injury (Tu et al., 2017). In animal models, blocking the TGF-β signaling pathway inhibits the occurrence of fibrosis (Argentou et al., 2016). In a clinical trial, TGF-β signaling was activated in patients with chronic HBV infection and suppressed by SMAD7 overexpression after successful antiviral treatment. Therefore, SMAD7 induction likely represents a candidate for novel therapeutic approaches (Argentou et al., 2016) (**Table 2** and **Figure 2**).

## Therapeutics That Modulate cGAS-STING Signaling Pathways to Control HBV Replication

Cyclic guanosine monophosphate–adenosine monophosphate (GMP-AMP) synthase (cGAS), considered a PRR and direct cytoplasmic dsDNA sensor. When cGAS binds to dsDNA, the cGAS-STING signaling pathway is activated and then induces the expression of IFN-I and other inflammatory cytokines, triggering innate immune responses (Barber, 2015). Because STING is widely expressed in various cell types and can regulate different programmed cell death pathways, a deeper understanding of the cGAS–STING signaling pathway could lead to a new light for treating infections, chronic inflammatory diseases, and even cancer (Chen et al., 2021a). Verrier et al. showed that HBV infection could suppress the expression of cGAS and its effector gene in both cell and mouse models (Verrier et al., 2018). Stimulator of IFN genes (STING; also known as MITA and MPYS, and encoded by TMEM173) is a signaling molecule associated with the endoplasmic reticulum (ER) and is essential for controlling the transcription of numerous host defense genes, including IFN-I and pro-inflammatory cytokines, following the recognition of aberrant DNA species or cyclic dinucleotides (CDNs) in the cytosol of the cell (Barber, 2015). Here, STING recruits and activates TANK-binding kinase 1 (TBK1) and IRF3 through serial phosphorylation events. NF-κB is also activated by STING in a TBK1-dependent manner in response to cytosolic dsDNA and collaborates with IRF3 to mediate dsDNA-induced gene expression of type I IFN (Khoo and Chen, 2018). To overcome the problem of low hepatocyte STING expression and HBV DNA cloaking (Guo et al., 2015), potential therapeutic agents for cGAS-STING signaling in liver diseases are developing. In preclinical trials, Gv1001 and daunorubicin can be used to inhibit the replication of HBV by eliciting mitochondrial stress and hepatocyte DNA damage, respectively (Imai et al., 2018; Choi et al., 2020; Chen et al., 2021b). Gv1001 and daunorubicin inhibit the replication of HBV by eliciting mitochondrial stress (mtDNA) and hepatocyte DNA damage (dsDNA) and develop inhibition of cccDNA, pgRNA, and nucleocapsid formation (Khoo and Chen, 2018). The cGAS-STING signals generate IL-6, IFN-I, and IFN-III to prevent HBV from evasion of IFN-I-induced cell response (Guo et al., 2015; Dansako et al., 2019; Choi et al., 2020). Thereby, the agonist of the cGAS-STING signaling pathway can be used in liver disease treatment, providing a new idea for understanding and treating liver diseases. cGAS-STING signaling activation also induces the expression of ISG56 (IFN-stimulated gene 56), which directly impairs HBV assembly and inhibits HBV RNA synthesis (Dansako et al., 2016). Altogether, the above studies support the mechanism that activating the cGAS-STING signaling pathway can significantly inhibit HBV replication *in vivo* (Chen et al., 2021a) (**Table 2** and **Figure 2**).

## Therapeutics That Modulate PI3K/AKT Signaling Pathways to Control HBV Replication

Many studies have shown that the HBV entry process might activate cellular signaling pathways. HBx can stimulate the PI3K/AKT signaling pathway in hepatocyte models, reduce HBV replication and increase HBV mRNA and core protein expression (Rawat and Bouchard, 2015). In addition, HBx also plays a critical role in activating signals and inhibiting hepatocyte apoptosis at the expense of higher levels of HBV replication (Rawat and Bouchard, 2015). Wang et al. demonstrated that HBxAg suppresses apoptosis of human placental trophoblastic cell lines *via* activating the PI3K/Akt signaling pathway (Wang et al., 2016). Consequently, the effects of HBx on apoptosis may be important for the establishment of HBV chronic infection and the development of HCC (Arbuthnot et al., 2000 ). AKT-regulated factors may provide therapeutic targets for inhibiting HBV replication (Rawat and Bouchard, 2015). It has been shown that prolonged treatment with PI3K-AKT-mechanistic target of rapamycin (mTOR) signaling pathway inhibitors markedly promote HBV copies in HBV replication and natural infection models (Xiang and Wang, 2018). The PI3K-AKT-mTOR pathway is therefore identified to be a negative regulator of HBV replication. These inhibitors enhance the replication and transcription of HBV in an HBx-dependent way. The results counterintuitively suggest that a PI3K inhibitor, Ly294002, inhibits the secretion of the small surface antigens of HBV in a PI3K-AKT-independent manner. Thus, the inhibitor Ly294002 can be developed as a drug against surface Ag secretion inhibitors (Xiang and Wang, 2018).

Autophagy and tripeptidyl peptidase II (TPPII) are associated with HBV infection. Tan’s study showed that adenovirus vector-HBcAg-TPPII promotes autophagy of CD8^+^ T cells, and inhibits HBV DNA replication and HBsAg expression in HBV transgenic mice, and elucidated that the PI3K/AKT signaling pathway may be involved in this autophagy process, and this theoretical basis provides a potential candidate for HBV immunotherapy (Tan et al., 2021). A similar effect was found on immature dendritic cells (imDCs) stimulated by nicotine. Nicotine-stimulated DCs induce HBV-specific cytotoxic T lymphocyte (CTL) priming by activating the PI3K/AKT pathway *in vivo* (Jin et al., 2012) (**Table 2** and **Figure 2**).

## Therapeutics That Modulate JAK/STAT Signaling Pathways to Control HBV Replication

During HBV infection, it has been shown that matrix metalloproteinase 9 (MMP-9) facilitates HBV replication by repressing the IFN/JAK/STAT pathway, IFN action, STAT1/2 phosphorylation, and IFN-stimulated gene (ISG) expression (Chen et al., 2017). Therefore, regulating the JAK/STAT pathway may develop novel molecular therapeutics against HBV replication. HBV encodes a microRNA (HBV-miRNA-3) that activates the JAK/STAT signaling pathway by down-regulating the inhibitory factor of cytokine 5 (SOCS5) in hepatocytes, thereby influencing host innate immunity to regulate HBV replication, enhancing the anti-HBV effect induced by IFN-I (Zhao et al., 2020). A novel liver-targeted interferon (IFN-CSP) (Lu et al., 2015), IFN-like small chemical compound CDM-3008 (Furutani et al., 2019), IFN-λ3 (Makjaroen et al., 2018), betaine (Zhang et al., 2016) and Tapasin (Wu et al., 2018) have significant activities in suppressing HBV replication through the JAK/STAT pathway *in vitro* and *in vivo* (**Table 2** and **Figure 2**).

## Therapeutics That Modulate RAS/MEK/ERK Signaling Pathways to Control HBV Replication

RAS/MEK/ERK (also known as the MAPK pathway) axis is recognized as the downstream pathway of vascular endothelial growth factor receptor 2 (VEGFR2). RAS is the first intracellular effector of the MEK/ERK pathway, and ERK is the main substrate of MEK (Zhao et al., 2021). Polyguluronate sulfate (PGS) enhances the secretion of IFN-β by upregulating the NF-κB and RAF/MEK/ERK pathways, which effectively inhibits the expression and secretion of HBsAg and HBeAg in HepG2.2.15 cells, as well as the replication of HBV (Wu et al., 2016). PGS could bind to and enter HepG2.2.15 cells to interfere with HBV transcription rather than block viral DNA replication. PGS as a novel anti-HBV agent deserves further investigation to modulate the host innate immune system in the future.

In preclinical trials, studies demonstrated that MLN4924 could block neddylation and activates the ERK signaling pathway to inhibit the expression of several transcription factors required for HBV replication (Xie et al., 2021), and the Forkhead box O 4 (FoxO4) transcription factor plays an important role in inhibiting HBV core promoter activity through ERK-mediated hepatocyte nuclear factor-4α (HNF4α) down-regulation *in vivo* (Li et al., 2019a). These agents may provide novel antiviral therapies against HBV infection (**Table 2** and **Figure 2**).

## Therapeutics That Modulate Chemokine Signaling Pathways to Control HBV Replication *In Vivo*

**Figure 3:**
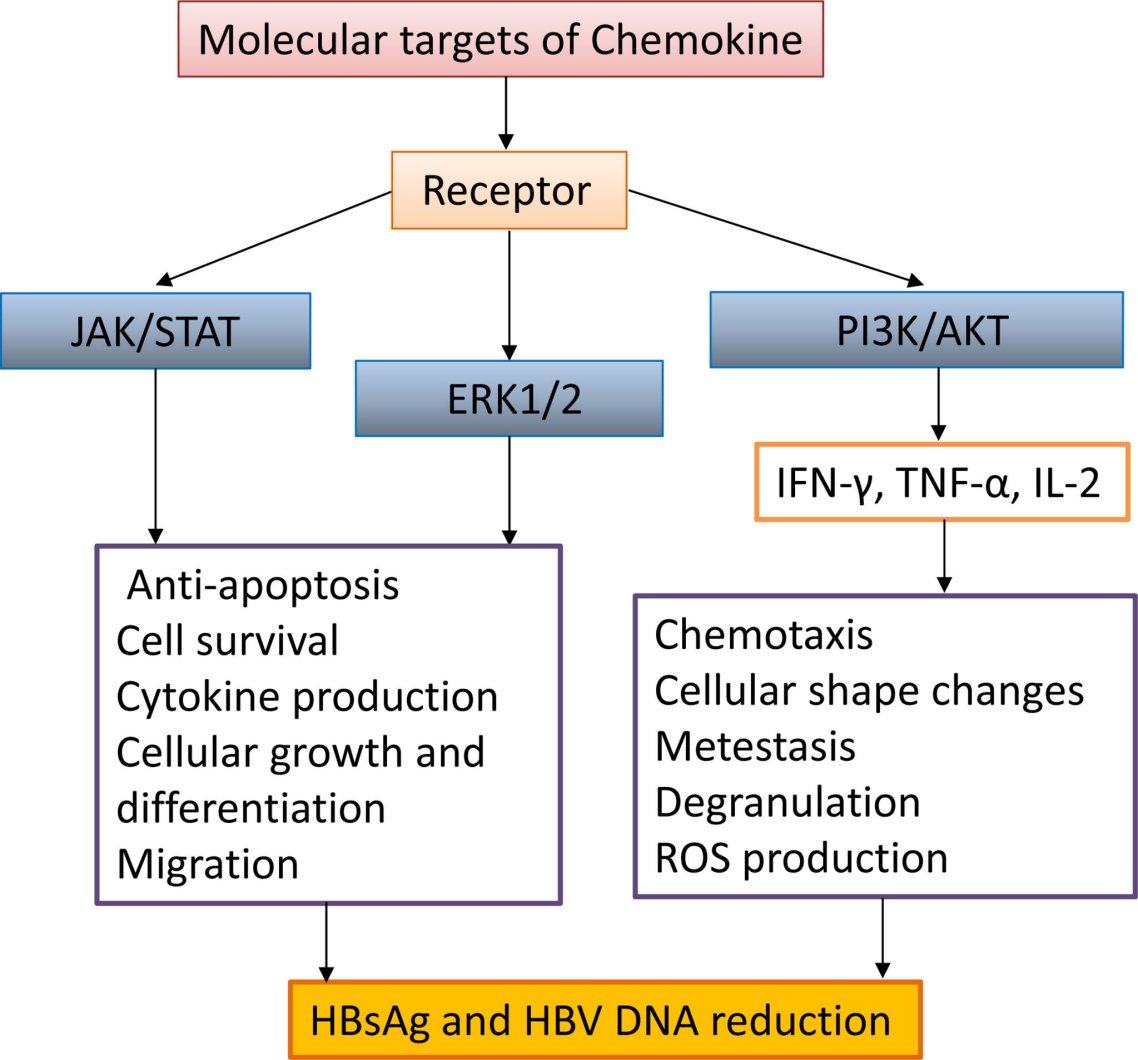
The interaction between chemokine signaling pathways and HBV infection. Chemokines activate intracellular JAK/STAT, ERK1/2, and PI3K/AKT signaling pathways, promote the secretion of antiviral proteins (IFN-γ, TNF-α, IL-2), promote activated cell migration, cell morphology change, survival, anti-apoptosis, and decrease HBsAg and HBV DNA.

Previous studies have shown that the trafficking of myelin DC cells (mDCs), antigen-presenting cells (APCs), and T cells are regulated by chemokine receptor expression and chemokine responsiveness (Palomino and Marti, 2015; Yan et al., 2019). There is growing evidence that certain chemokines in the liver, such as chemokine (C-X-C motif) ligand 13 (CXCL13) and chemokine (C-C motif) ligand 19 (CCL19), are necessary for naïve lymphocyte activation and expansion in the HBV clearance and provide an appropriate environment (Liu et al., 2017; Yan et al., 2021). In the context of chronic HBV infection, mDCs are more efficient than other APCs (Matsuno et al., 1996). Upon migrating, hepatic mDCs upregulate C-C chemokine receptor type 7 (CCR7) expression and increase its responsiveness to CCL19 (Abe et al., 2004). Many studies have demonstrated that hepatic mDCs play a critical role in promoting immune tolerance by producing IL-10 and TGF-β, activating regulatory T (T_reg_) cells or regulatory B (B_reg_) cells, and suppressing effector T (T_eff_) cell proliferation (Abe et al., 2004; Liu et al., 2018). Hepatocytes’ immune tolerance is a major factor in the formation of HBV chronic infections. In acute inflammation, mDCs show a non-immune tolerance phenotype, which helps to promote the activation of T cells (Abe et al., 2004). However, some chemokines can activate immune cells in the liver. For example, CCL19 plays an important role in activating intrahepatic cells, such as CD8^+^ T cells, and promoting HBV clearance in the CHB mouse model (Yan et al., 2019; Yan et al., 2021). In CHB patients, high expression of follicular helper T (Tfh) cell and CD4+ T cell C-X-C chemokine receptor type 5 (CXCR5) has been identified as positively correlated with immune activation. However, the understanding of the increased expression of chemokine receptors is limited (Huang et al., 2018). It has been shown that HBV infection does not affect the overall frequency of CD8^+^ CXCR5^+^ cells, while chronically infected patients accompanied with CD8^+^ CXCR5^-^ cells increased. In addition, the number of CD8^+^ CXCR5^+^ T cells was significantly associated with a decrease in HBsAg and HBV-DNA and the production of HBV-specific cytokines, although accompanied by an increase in PD-1 expression (Khanam et al., 2021). Altogether, there is considerable knowledge about the molecular virology of viral proteins that provide highly efficient strategies to modulate and prevent apoptotic signals’ transduction and favor virus infection, such as the HBx gene of HBV (Hauser and Legler, 2016; Shin et al., 2016). We have searched CCR7-associated chemokine signaling pathways on KEGG PATHWAY Database and discussed the multiple regulatory mechanisms of chemokine signaling and the impact on the downstream function of CCR7 during viral infection. (Yan et al., 2019) (**Table 2** and **Figure 3**).

## Conclusions

Treatment options for chronic HBV infection remain limited, and some of the available therapies can produce drug-resistant HBV mutants. Since the rarity of the complete treatment response and the difficulty of obtaining liver biopsy specimens from CHB patients have hindered the recognition of the characterization of immune determinants of viral control, animal models are widely used to identify potential mechanisms of HBV clearance. As a result, there is continuing interest in understanding the molecular mechanisms that regulate HBV replication, and new molecular agents are constantly being pushed into clinical trials. In addition, intracellular molecules associated with the development of chronic HBV infection can serve as potential therapeutic targets.

Altogether, this review suggests that clinical studies on the effects of signaling molecular interventions on HBV replication other than TLR agonists are still relatively rare and that studies of some PRR signaling molecules are still at preclinical levels and limited in number. Therefore, novel drugs targeting cell signaling pathways to control HBV infection must be explored in more CHB animal models or patients.

## Author Contributions

YY, YQ, CD, and CZ conceived the paper and generated the figures. All authors read and approved the final manuscript.

## Funding

This work was funded by the National Natural Science Foundation of China (81701550), the Top Talent Support Program for young and middle-aged people of Wuxi Health Committee (BJ2020094), the Wuxi Key Medical Talents Program (ZDRC024).

## Conflict of Interest

The authors declare that the research was conducted in the absence of any commercial or financial relationships that could be construed as a potential conflict of interest.

## Publisher’s Note

All claims expressed in this article are solely those of the authors and do not necessarily represent those of their affiliated organizations, or those of the publisher, the editors and the reviewers. Any product that may be evaluated in this article, or claim that may be made by its manufacturer, is not guaranteed or endorsed by the publisher.

## Abbreviations

HBV, hepatitis B virus; HCC, hepatocellular carcinoma; HBxAg, HBV x antigen; HBsAg, HBV surface antigen; HBeAg, HBV e antigen; cccDNA, covalently closed circular DNA; CHB, chronic hepatitis B; IRF3, interferon regulatory transcription factor 3; miRNA, microRNA; TLR, toll-like receptor; CKR, chemokine receptor; HCV, hepatitis C virus; TNF-α, tumor necrosis factor-α; IFN, interferon; NF-κB, nuclear factor kappa B; ERK, extracellular signal-regulated kinase; MAPK, mitogen-activated protein kinase; PI3K/AKT, phosphatidylinositol 3-kinases/protein-serine-threonine kinase; PRR, pattern recognition receptor; peg-IFN-α, pegylated-interferon alfa; TNF-α, tumor necrosis factor-a; IFN-α, interferons-α; LPS, lipopolysaccharide; WHV, woodchuck hepatitis virus; pDCs, plasmacytoid dendritic cells; MyD88, myeloid differentiation response protein 88; JNK, c-Jun N-terminal protein kinase; RIG-I, Retinoic acid-inducible gene I; APC, antigen-presenting cell; mDC, myelin DC cell; TGF-β, transforming growth factor β; cGAS, cyclic guanosine monophosphate–adenosine monophosphate synthase; mTOR, mechanistic target of rapamycin; VEGFR2, vascular endothelial growth factor receptor 2; JAK/STAT, Janus family tyrosine kinases/signal transducer and activators of transcription; AP, activating protein; CTL, cytotoxic T lymphocyte; NFATc3, nuclear factor of activated T cells 3; HNF4α, hepatocyte nuclear factor-4α; CCL19, chemokine (C-C motif) ligand 19; CCR7, C-C chemokine receptor type 7; CXCL13, chemokine(C-X-C motif) ligand 13; CXCR5, C-X-C chemokine receptor type 5; Tfh cell, follicular helper T cell.
