# Corrigendum: Novel molecular therapeutics targeting signaling pathway to control hepatitis B viral infection

**DOI:** 10.3389/fcimb.2023.1253110

**Published:** 2023-07-21

**Authors:** Yan Yan, Yuanwang Qiu, Chantsalmaa Davgadorj, Chunfu Zheng

**Affiliations:** ^1^ Laboratory for Infection and Immunity, Hepatology Institute of Wuxi, The Fifth People’s Hospital of Wuxi, Affiliated Hospital of Jiangnan University, Wuxi, China; ^2^ Department of Microbiology, Immunology and Infectious Diseases, University of Calgary, Calgary, AB, Canada

**Keywords:** HBV, TLR, carcinogenesis, signaling pathway, therapeutics, infection

In the published article, there was an error in the affiliations. Instead of three, there are only two affiliations. The original affiliation 2 was removed. The correct affiliations are listed below:

Instead of

Yan Yan ^1^, Yuanwang Qiu ^1^, Chantsalmaa Davgadorj ^1^, Chunfu Zheng ^2,3^

“^1^Laboratory for Infection and Immunity, Hepatology Institute of Wuxi, The Fifth People’s Hospital of Wuxi, Affiliated Hospital of Jiangnan University, Wuxi, China.
^2^Department of Immunology, School of Basic Medical Sciences, Fujian Medical University, Fuzhou, China.
^3^Department of Microbiology, Immunology and Infectious Diseases, University of Calgary, Calgary, AB, Canada.”, it should be

Yan Yan ^1^, Yuanwang Qiu ^1^, Chantsalmaa Davgadorj ^1^, Chunfu Zheng ^2^

“^1^Laboratory for Infection and Immunity, Hepatology Institute of Wuxi, The Fifth People’s Hospital of Wuxi, Affiliated Hospital of Jiangnan University, Wuxi, China.
^2^Department of Microbiology, Immunology and Infectious Diseases, University of Calgary, Calgary, AB, Canada.”

The authors apologize for this error and state that this does not change the scientific conclusions of the article in any way. The original article has been updated.

